# Challenges and Solutions for Global Water Scarcity

**DOI:** 10.3390/membranes13060612

**Published:** 2023-06-20

**Authors:** Hilla Shemer, Shlomo Wald, Raphael Semiat

**Affiliations:** 1 The Wolfson Department of Chemical Engineering, Technion-Israel Institute of Technology, Haifa 3200003, Israel; shilla@technion.ac.il; 2 Wald Industries, Tor HaAviv 1, Rehovot 7632101, Israel; shlomo.wald@gmail.com

**Keywords:** desalination, reverse osmosis, wastewater reclamation, energy consumption, environmental impacts

## Abstract

Climate change, global population growth, and rising standards of living have put immense strain on natural resources, resulting in the unsecured availability of water as an existential resource. Access to high-quality drinking water is crucial for daily life, food production, industry, and nature. However, the demand for freshwater resources exceeds the available supply, making it essential to utilize all alternative water resources such as the desalination of brackish water, seawater, and wastewater. Reverse osmosis desalination is a highly efficient method to increase water supplies and make clean, affordable water accessible to millions of people. However, to ensure universal access to water, various measures need to be implemented, including centralized governance, educational campaigns, improvements in water catchment and harvesting technologies, infrastructure development, irrigation and agricultural practices, pollution control, investments in novel water technologies, and transboundary water cooperation. This paper provides a comprehensive overview of measures for utilizing alternative water sources, with particular emphasis on seawater desalination and wastewater reclamation techniques. In particular, membrane-based technologies are critically reviewed, with a focus on their energy consumption, costs, and environmental impacts.

## 1. Introduction

Globally, more than 40% of the population experiences water scarcity, with over 700 million people lacking access to clean drinking water [1]. Approximately 50% of human-generated wastewater is discharged directly into rivers or oceans without any treatment, causing severe environmental and health consequences. The lack of safe and reliable drinking water may lead to desertification, forced migration, hunger, diseases, and domestic or regional conflicts. For instance, the Atatürk Dam on the Euphrates River has enabled extensive irrigation within Turkey, while reducing water quantity and quality in Iraq and northeastern Syria [2]. It is important to note that the impact of water scarcity in developing countries is more challenging than in Western countries. To create a sustainable water infrastructure, the focus should be on conservation, protecting water sources, and limiting pollution.

Desalination is considered one of the most effective ways to increase water supply and provide clean and affordable water to millions of people. Nevertheless, it is important to recognize that addressing the global water crisis requires a multifaceted approach. In addition to desalination, other measures should be implemented, such as centralized governance, education, improvements in water catchment and harvesting technologies, irrigation, agricultural practices, distribution infrastructure, prevention of leakage, wastewater reclamation, pollution treatment and prevention, investment in innovative technology, and transboundary water cooperation.

This review is divided into three sections. The first section provides a brief overview of the measures required to address the global water shortage. The second section focuses on membrane-based seawater desalination, which accounts for approximately 80% of the world’s total desalination capacity, equivalent to approximately 90 Mm^3^/d. This technology is also the preferred option for most new desalination facilities [3]. The third section concentrates on membrane-based wastewater reclamation.

## 2. Essential Measures to Address the Worldwide Water Shortage

### 2.1. Centralized Governance

Centralized governance is essential for providing guidelines in overseeing all water-related issues to ensure adequate water supply. A key aspect of such a system is a water authority which holds the responsibility of licensing the drilling of wells, desalinating water, treating domestic and industrial wastewater, designing and constructing water infrastructure, and setting water prices [4,5,6].

The government must adopt recommendations to achieve progress in water management. However, it is not feasible for the government to accomplish this task solely by itself [7]. Collaborative water governance can enhance the sustainability, equity, and efficiency of water management by capitalizing on the expertise, knowledge, and resources of all stakeholders. Collaborative water governance is a multi-stakeholder approach involving various entities such as government agencies, non-governmental organizations, community groups, and private sector entities, working in conjunction to manage water resources and supply [8]. This approach provides a comprehensive understanding of water needs at the national and sub-national levels, considering both present and future needs, as well as considering potential extreme circumstances, such as droughts and floods, which can significantly affect water availability and quality. The development of contingency plans that outline appropriate actions during such events will help to mitigate their impact and ensure the continuity of the water supply.

### 2.2. Education

It is imperative to raise awareness about the water scarcity issue and encourage individuals, organizations, and governments to take action. Promoting an understanding of the value of water and the significance of its protection at national and international levels through educating children and communities is of utmost importance. Water conservation measures must include both household and industrial water usage, with the latter accounting for approximately 22% of total global water consumption [9].

Private households can implement various water conservation techniques to minimize water usage, such as using water-saving faucets and toilets, collecting rainwater for garden irrigation, and growing low-water-demanding plants. On the other hand, industrial water conservation measures comprise adopting water-saving manufacturing processes and reusing wastewater. For instance, an effective approach is to replace once-through cooling systems with closed-circuit cooling systems, where only the evaporated water is replenished [10]. 

### 2.3. Water Catchment and Harvesting Technologies

Water harvesting and catchment technologies vary depending on the water source, i.e., surface water, rainwater, floodwater, and groundwater. These technologies are utilized in a multitude of settings, including domestic, industrial, and agricultural sites, as well as wetlands. Some examples of these technologies are rainwater harvesting aquifer recharge, floodwater harvesting systems, and dams [11]. Rainwater harvesting (RWH) is the most traditional and sustainable method of water harvesting. The key advantages of RWH systems are that they can augment the water supply, provide an alternative to potable water for non-potable uses, and help reduce storm water runoff [12]. Nevertheless, concerns have been raised regarding its feasibility, primarily due to the quality of rainwater that is dependent on airborne components. As a result, the successful implementation of rainwater harvesting depends on effective treatment to eliminate contaminants [13]. In addition, RWH systems have limited water supply potential and are substantially more expensive than centralized systems [14]. Recent review articles on RWH are available [12,15,16,17,18,19,20,21,22].

In addition to increasing groundwater levels [23,24,25], aquifer recharge can also mitigate soil erosion caused by runoff, and distribute water availability more evenly throughout the year. Moreover, it can reduce the risks associated with hydrometeorological events such as drought and flooding, promote soil moisture, and regulate water tables that support vegetation and biodiversity [26]. However, the successful implementation of community-based aquifer recharge strategies requires a thorough understanding of the socio-hydrological system in which they are implemented [25].

### 2.4. Water Infrastructure

Strong water infrastructure is crucial for ensuring a reliable water supply, reducing waste, improving quality of life, and preventing the spread of water-borne diseases. There are several challenges to water infrastructure including aging, improper maintenance, and cyber-physical threats. As infrastructure and equipment age, they require repairs, overhauls, or replacements. However, establishing a sustainable infrastructure necessitates a significant financial investment to maintain critical parts and networks in operational conditions [27]. 

Inadequate maintenance of water infrastructure may cause its components to deteriorate or be damaged over time, which can compromise the quality of water and result in interruptions to service [28]. Therefore, it is essential to recognize proper infrastructure management and maintenance as a significant issue in water management practice [29]. 

In the water management context, Cyber-Physical Systems (CPS) are designed to integrate physical water assets with networked devices, enabling the monitoring and control of various physical processes in water and wastewater treatment plants and distribution systems. The primary objective of CPS in water management is to minimize leakage, guarantee water quality, and optimize operational efficiency [30]. While CPS technologies offer significant benefits, they also introduce cyber-physical threats that can potentially compromise the safety and reliability of the water supply. Thus, it is crucial to develop effective strategies that can detect and mitigate both cyber and physical threats, which may result in damage to physical components, (pumps, valves, and tanks) as well as the overall water supply and quality [31].

### 2.5. Irrigation and Agricultural Practices

The agricultural industry has been recognized as a major consumer of water resources, accounting for 70% of the world’s freshwater withdrawal to irrigate approximately a quarter of the world’s cropland. Due to the combined effects of climate change and population growth, water availability for agricultural production is becoming increasingly scarce. To address these challenges, water management approaches need to be adopted, along with precision agricultural technologies to enhance water use efficiency and satisfy the requirements of agricultural production, despite the diminishing availability of land and water resources [32]. Precision agriculture and smart irrigation technologies enable farmers to optimize resource use and prevent plant water stress. Smart irrigation involves monitoring and controlling strategies to supply water at the appropriate time, location, and quantity, considering soil moisture, weather patterns, and plant conditions. Traditional irrigation methods can lead to over- or under-irrigation, resulting in nutrient leaching, water waste, and quality and yield loss [33].

Enhancing irrigation efficiency is crucial for minimizing water usage in agricultural operations while maintaining maximum crop yields and reducing environmental impacts [34]. Enhanced irrigation can be achieved through the adoption of effective irrigation systems such as drip irrigation or deficit irrigation, improvement in the precision of water application, changes in farming practices such as crop rotation and conservation tillage, and the repair of irrigation system leaks or damages. These strategies can significantly reduce the carbon footprint, limit water consumption, minimize agricultural runoff, decrease energy requirements for water pumping and transportation, and reduce irrigation-related costs for extraction and transport [35].

Drip irrigation is important in sustainable agriculture for its precise delivery of water and nutrients to plant roots. However, it has challenges such as clogging and soil salinization. Subsurface irrigation is a viable alternative to address these issues, reducing evaporation and weed growth, and increasing safety with reclaimed wastewater. Desalination of effluent also address the problem of salinity buildup [36]. The injection of fertilizers into the water stream of a drip irrigation system (i.e., fertigation) enables precise applications of nutrients, which can lead to higher crop yields and improved soil health. 

### 2.6. Pollution Control

Water pollution poses risks to ecological and human health. Inorganic and organic pollutants, along with microbial agents, are increasingly seen as harmful to ecosystems and organisms. Therefore, it is necessary to allocate resources to mitigate water pollution. Emerging contaminants (ECs) such as pharmaceuticals, personal care products, plasticizers, surfactants, fire retardants, nanomaterials, and pesticides have received attention in recent years due to their hazardous effects. Physical, chemical, and biological techniques are being explored to remove ECs and reduce their harmful effects. The scientific literature comprehensively reviews these techniques [37,38,39,40,41,42,43,44,45,46]. 

### 2.7. Novel Technologies

Developing effective, low-cost, and robust technologies for water and wastewater treatment is critical for improving sustainable water production and management [47]. To attain sustainable technological change, it is imperative to recognize that progress in technology alone is insufficient. It is equally important to account for the economic and societal factors that can impact the success and longevity of technological advancements [48]. 

Numerous obstacles hinder innovation within the water industry. One such barrier is the high initial costs and long lifespan of existing infrastructure, leaving minimal flexibility for the rapid integration of newer technologies. Additionally, the low cost of water results in less funding for future investments. Moreover, public health risks associated with water systems limits the ability to take risks in the implementation of new approaches. Finally, existing regulations may not facilitate innovation in the water industry, and even when they do, water utilities often do not prioritize research and development efforts [49].

While novel techniques with promising feasibility on a laboratory scale exist, they often face challenges such as low process efficiency, high-energy requirements, a lack of engineering expertise for scaling up, low economic benefits, and poor infrastructure. There are also gaps in innovation that exist between conceptual ideas and solutions that are ready for scale-up [50]. In addition, due to their relative specificity, novel technologies often pose a challenge in terms of adaptability to different needs once they have been developed [51]. 

To overcome all of the above-mentioned challenges, it is crucial to focus on the organizational culture of water management entities and encourage the integration of innovative solutions. Policies should promote the development of services that prioritize innovation and adaptation to address water-related challenges and stimulate economic growth. Several factors that foster innovation in the water industry include a supportive culture that values innovation, regulations that encourage innovation, adequate financial resources for research and development, and crucially, public support [49].

### 2.8. Transboundary Water Cooperation 

Due to the intricate interplay of diverse economies, ecosystems, climates, politics, and cultures within watersheds, managing transboundary water resources is essential [52]. It involves cooperation, coordination, and joint action between countries. Securing the availability of water faces significant challenges, such as concerns about the loss of national sovereignty, misunderstandings about the risks and benefits of collaboration, and a lack of capacity and political will [53]. 

Collaboration on transboundary water management has been shown to yield positive effects, such as increased energy and food production, improved disaster resilience, enhanced economic connectivity [54], and potentially, environmental sustainability and political stability [53]. Table 1 presents the benefits of such collaboration, categorized into economic, societal, and environmental advantages.

The Jordan-Israel energy and water agreement is a prominent example of transboundary water cooperation. It involves building a large solar farm in Jordan that would supply power to Israel in exchange for water. Additionally, a new desalination plant will be constructed on the north Mediterranean coast of Israel to supply water to both Jordan and Israel. Cooperation also extends to the southern region of the two countries, where a new desalination plant will be built to provide water to both nations.

Key components for effective transboundary water management include financing, exchange of information, enforcement [55], equitable access, responsibility and transparency, stakeholder participation, and inclusiveness [56].

## 3. Desalination

### 3.1. Overview

Membrane-based technologies are employed for water and wastewater treatment, desalination, production of value-added materials, biofuel, and use in the food industry. The primary methods of membrane filtration are microfiltration (MF), ultrafiltration (UF), nanofiltration (NF), and reverse osmosis (RO). Integration of two or more of these methods is applied to address the limitations of each process. Despite their many benefits, membrane-based technologies face significant challenges in the form of public perception regarding high-energy consumption, environmental impact, and cost [57].

The desalination industry has become a reliable source of freshwater supply in many countries, with applications including seawater (SW), brackish water (BW), and wastewater (WW) desalination. Despite its steady growth, the challenge persists of lowering costs to enable widespread use for drinking water supply and the safe reuse of wastewater for agricultural irrigation and other purposes. Evaporation and condensation technologies were the first desalination techniques historically applied. These include multi-effect distillation (MED), multi-stage flash (MSF) desalination, membrane distillation, and thermal vapor compression. Newer techniques include solar still distillation and humidification-dehumidification desalination [58], freezing desalination [59], and capacitive deionization [60,61]. Membrane-based desalination includes RO, electrodialysis (ED), electrodialysis reversal (EDR), NF, and forward osmosis. This section focuses on seawater reverse osmosis desalination, as it accounts for approximately 80% of the world’s total desalination capacity [3].

In 2020, the total desalination capacity worldwide was approximately 115 Mm^3^/day (42 billion m^3^/year) [3]. Early desalination plants predominantly utilized thermal technologies, especially in the Middle East. Since the adoption of reverse osmosis technology in the 1980s, the dominance of thermal technology has gradually decreased. In 2000, the capacity of desalinated water produced by thermal technologies (mainly MSF) and RO was about the same at 11.6 and 11.4 Mm^3^/d, respectively, accounting for 93% of the total world capacity of desalinated water. While thermal technology has only slightly advanced since 2000, the number and capacity of RO plants have grown exponentially [3].

The composition of saline waters varies widely. These waters contain dissolved inorganic and organic compounds, silt, colloidal suspensions, nanoparticles, viruses, bacteria, and other impurities. Though desalination processes separate salts, other treatment technologies are often necessary for the removal of specific impurities such as boron and silica. Boron is a vital element for organic growth at concentrations < 1 mg/L, while higher levels may harm plants. Despite the World Health Organization (WHO) recommendations of 2.4 mg/L of boron in desalinated water [62], some countries require a lower maximum concentration. In Israel, for example, the regulations are set at 0.3 mg/L [63] since the reclaimed waters are used for irrigation purposes. To improve boron rejection in desalination processes, a variety of techniques are used, including a second RO pass, which often involves a pH change on the feed side of the membrane. Other methods for boron removal include ion exchange [64] and ED [65]. In some cases, integration of these techniques may be used. Mitigation of silica scaling is achieved by eliminating silica or hardness ions from the feed water through techniques such as lime softening, coagulation, electro-coagulation, adsorption, ion exchange, and seeded precipitation [66]. The most commonly employed pre-treatment method is the use of ion exchange softeners to remove hardness ions. This method is typically combined with pH adjustment to reduce silica scaling potential [67].

Desalination has the potential to offer viable solutions to water scarcity, especially in countries with proximity to oceans and seas. In the case of inland and remote communities, desalination can be utilized to recover low-quality water to the greatest extent feasible.

### 3.2. Seawater Reverse Osmosis

**Figure 1:**
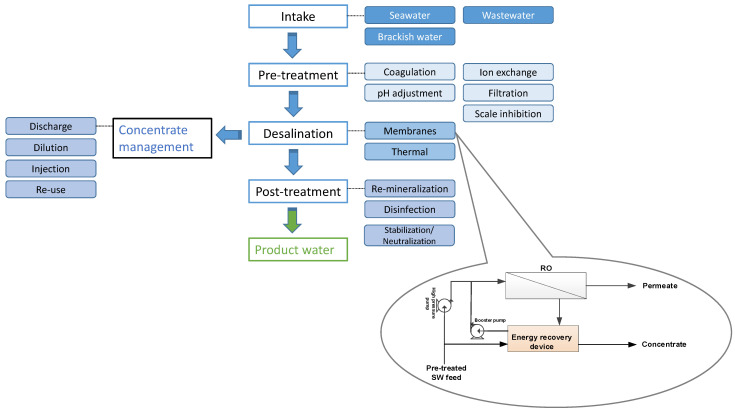
Schematics of the desalination process.

In the RO desalination process, saline water is subjected to a pressure higher than the osmotic pressure while being exposed to a selectively permeable membrane. This allows the permeation of fresh water through the membrane while rejecting the dissolved salts. The desalination process involves a series of process trains that encompass a feed intake system, feed water pretreatment, desalination separation system, energy recovery devices, product water post-treatment, and brine management (Figure 1). Technical reliability, simplicity, large-scale continuous operation, economic viability, low energy consumption, and environmental safety are essential criteria for determining the feasibility of a desalination process. 

A water intake system is imperative for providing raw feed water from its source to the plant. Pre-treatment of the raw RO feed water aims to reduce the levels of suspended solids (SS), organics, and microorganisms to mitigate membrane fouling and scaling. As the purified water permeates through the membrane module, there is a gradual increase in the concentration of all dissolved species. When the solubility limits of sparingly soluble salts, such as CaCO_3_ and CaSO_4_, are exceeded, it leads to the precipitation of a scale layer on the membrane. Scale deposition is challenging to tolerate due to its highly deleterious effects on production capacity and specific energy consumption. Fouling comprises sedimented particulates, organic matter, and biofilms. The degree of fouling/scaling depends, among other factors, on the membrane properties, hydrodynamics, and water quality [68]. Pre-treatment methods, such as flocculation/coagulation, disinfection, media filtration, ultrafiltration, or microfiltration, are applied to minimize the effects of scaling/fouling species [69,70].

Antiscalants can extend the effective solubility limits of scaling salts, enabling a viable water recovery fraction i.e., the product-to-feed ratio. The conspicuous advantage of antiscalants is their ability to suppress precipitation from supersaturated solutions at very low dosages (typically below 10 mg/L), making them an affordable solution [71]. By suppressing mineral crystal nucleation and growth and promoting crystal dispersion, antiscalants prevent scaling. Other measures to prevent scaling include pH adjustment, periodic membrane cleaning, limiting water recovery, and removing scale precursors [72].

Desalinated water lacks minerals and, therefore, requires post-treatment to meet health standards and become non-corrosive and palatable. The specific post-treatment processes depend on water quality goals and may include disinfection, re-mineralization, and pH adjustment (i.e., neutralization/stabilization) [73,74]. 

Brine management is necessary to deal with the high salinity of the waste stream. Seawater reverse osmosis (SWRO) brine can be discharged through designated open channel outfalls, which involve mixing the brine with power plant cooling water or wastewater from a treatment plant, or through an offshore sub-sea multiport diffuser system [75]. The brine disposal methods for inland brackish water desalination plants comprise several options, including enhanced evaporation ponds, deep well injection, discharge to surface water or wastewater treatment plants, and using it for non-sensitive crop irrigation. The following review articles have recently been published on the topic of brine management and treatment options for achieving zero-liquid discharge (ZLD) or near-ZLD [76,77,78,79]. The selection of the appropriate disposal method is influenced by several factors such as brine volume and quality, the discharge point’s physical or geographical location, receiving site availability, environmental and public acceptability, and capital and operating costs. However, the brine also contains energy in the form of pressure, which can be efficiently recovered (up to 96%) by various energy recovery devices such as a pressure exchanger. The incorporation of energy recovery devices helps in reducing the energy consumption of the desalination process [80].

Water production in both brackish water reverse osmosis (BWRO) and SWRO plants is regulated by the recovery ratio. The water recovery in BWRO plants typically ranges from 70–95% at operating pressures of 10–25 bars, depending on the feed water’s hardness and silica content. In SWRO, the water recovery ratio is lower, ranging from 35–50% at operating pressures of 40–80 bars. The primary factors influencing the quality of the desalinated water are attributed to the design of the desalination system, the type of membrane utilized, chemical treatments, and post-treatment procedures [81]. Certain small molecules, including carbon dioxide, hydrogen sulfide, silica, and boric acid, can infiltrate through the membrane, causing a decrease in the product’s water quality. This can be resolved by aeration, ion exchanger, and/or mixing. Additionally, small organic compounds that are dissolved in the source water may also penetrate the product water [82].

#### Energy Demand and Cost of SWRO Desalination

Over the years, there have been significant technological advancements in the fields of membranes, membrane modules, plant standardization, operational efficiency, and energy recovery, resulting in a consistent decrease in the cost of seawater reverse osmosis technology [83]. Manufacturers of various components including membranes, pumps, pressure exchangers, valves, and controllers have invested considerable efforts towards enhancing their products by reducing their energy requirements and prolonging their operational lifespan.

The comprehensive cost of producing water in a typical RO desalination plant encompasses various factors, including land cost, energy consumption, equipment expenditure, membrane replacements, pre- and post-treatment expenses, brine management, labor charges, maintenance costs, and finance charges [72]. The cost breakdown varies significantly depending on the size and geographical location of the plant, the quality and salinity of the source water, and the prevailing electricity rates. It is crucial to recognize that water, despite being the most valuable commodity on earth, is also the cheapest.

**Figure 2:**
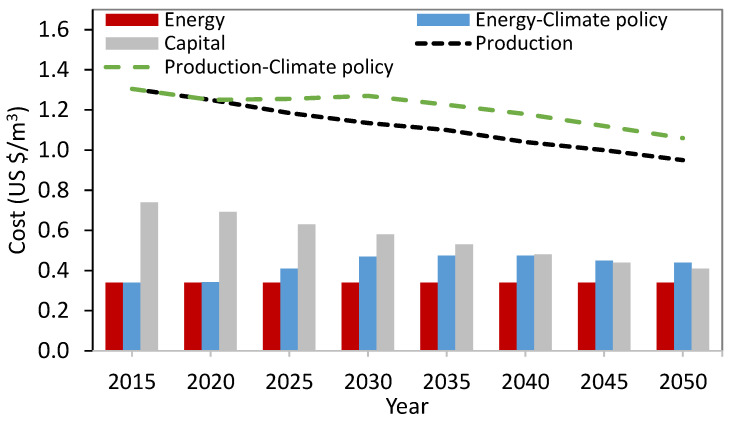
Averaged predicted unit production, capital, and energy costs of SWRO desalination. Climate policy assumed stringent policies to constrain global warming (data obtained from [84]).

It is anticipated that the cost of SWRO will continue to decrease, as displayed in Figure 2. Gao et al. [84] estimated the energy, capital, and production costs of SWRO desalination from 2015 to 2050. The authors validated their model by relying on the previous production costs of SWRO plants in 140 countries from 1990 to 2014. Afterward, they predicted average future costs on a global scale. Moreover, changes in socioeconomic situations and strict climate policies aimed at mitigating global warming were introduced, including renewable energy resources and a higher carbon tax than the current rate. As seen in Figure 2, a significant decrease in capital costs is anticipated, which can be attributed to technological advancements and economies of scale. Under conventional fossil fuel operations, energy costs are projected to remain unchanged, while they will increase primarily due to the elevated electricity rates brought on by climate policies. The projected production costs are anticipated to fall within the range of 1.30–0.94 and 1.30–1.10 US$/m^3^ under fossil fuels and climate policies, respectively [84].

The energy demands of a desalination plant are influenced by several factors, including the desalting technology, engineering, energy recovery, water recovery level, characteristics of the feed water, and quality requirements of the product water. It should be noted that the quality of the desalinated water has a direct impact on the quality of the treated wastewater, which is subsequently utilized for various applications. Field experience and scientific research have indicated that the energy consumed by SWRO desalination only accounts for a small fraction of the total national energy consumption [85,86]. Properly located, designed, and operated desalination plants can significantly minimize energy demand and the associated environmental impact.

The energy consumption for SWRO desalination ranges between 3.5–4.5 kWh/m^3^ [87], with the membrane separation process accounting for 71% of the total energy consumption [88]. Various approaches have been implemented to alleviate the energy consumption and costs of RO desalination. These include the adoption of technical advancements such as the improvement of energy recovery devices alongside the development of efficient high-pressure pumps; improved materials for the development of highly permeable and/or low-fouling composite membranes plus the extension of membrane lifespans; improved process design including optimization of operating conditions as well as process configurations alongside the use of integrated processes; and the use of renewable energy sources [83,86]. 

Digitalization involves monitoring, performance optimization, and fault prediction. The utilization of artificial intelligence algorithms and big data analytics can assist in optimizing the utilization of available data and information, leading to better decision-making and enhanced service delivery resulting in reduced operational costs [89]. It has also been argued that, in some cases, the energy costs of pumping or transporting water may be greater than the energy needed for water production by a large desalination plant located in proximity to water consumers, particularly when such a plant makes use of off-peak electricity [72].

The prices for desalinated water produced in a large SWRO desalination plant range from 0.28 to 0.53 US$/m^3^, for the plant’s capacity ranging from 909,000 to 545,000 m^3^/day [90]. For smaller desalination plants, the prices are higher, ranging from 0.48 to 1.72 US$/m^3^, for plants’ capacities of 6000 and 4800 m^3^/day, respectively [91]. In Israel, where close to 80% of the urban water supply is desalinated seawater, the total cost for a household customer is slightly above 2.2 US$/m^3^, which includes the cost of water transportation, wastewater treatment, and taxes. However, farmers pay about 1 US$/m^3^ for drinking water and around 0.35 US$/m^3^ for reclaimed wastewater. Historical information on RO cost can be found in references [92,93,94,95,96]. 

### 3.3. Environmental Aspects of SWRO Desalination 

Uncertainties and public concerns persist regarding the environmental impacts of desalination. These impacts comprise the construction stage, carbon footprint, and impact of brine discharge on marine and coastal environments. An additional environmental liability arises from aged RO membranes which have reached their end of life cycles. As of 2020, global desalination capacity stands at 115 Mm^3^/day, resulting in about 170.6 mM/m^3^ of brine, assuming the same ratio of product water to brine as found in [97]. Desalination projects undergo environmental impact assessments at every stage from planning through design, construction, and operation. This section focuses on the effect of brine discharge on the marine environment, which is a major concern due to the potential harm it can cause to marine life and ecosystems through increased salinity and toxicity levels. The effects of construction and carbon footprint are briefly discussed. 

The construction and operation of a desalination plant can have a range of environmental impacts, including alterations to the coastal environment, obstructing access, noise, vibrations, alterations to native fauna, sediment discharge into water bodies, and accidental spills or leaks of hazardous chemicals [98]. Since fossil fuels are the primary energy source for desalination, the process is directly associated with emissions of greenhouse gases and air pollution. The emission of CO_2_ in RO desalination is estimated at 0.6–4.3 kg CO_2_/m^3^ with a maximum value of 8.6 kg CO_2_/m^3^. It is worth mentioning that using off-peak electricity can reduce the carbon emissions of the electricity grid. 

The carbon footprint of desalination can be reduced by using alternative energy sources, such as nuclear, solar, and wind, which are estimated to emit only 0.14–0.19 kg CO_2_/m^3^ [99]. Some of the alternative energy sources are commercially available technologies, such as nuclear, wind, and hydroelectric power, while others remain in prototype form due to their high cost and inability to produce energy continuously [100]. However, the main obstacle to renewable energy sources is their intermittent character. Steady energy sources from renewable energy technologies can be achieved by feeding the electricity generated into the grid while the desalination plant uses the grid. In addition to the above-mentioned renewable energy sources, natural gas is considered to be more environmentally friendly when compared to other fossil fuels. Its favorable characteristics include reduced emissions, competitiveness with coal, and the ability to support the integration of renewables. These attributes make it a viable and sustainable option for transitioning to renewable energy on a global scale [101,102]. 

Desalination has potential impacts on the marine environment, including entrainment and impingement of marine organisms from seawater intake, brine discharge, and chemical disposal. Water intake designs are site-specific, so assessing physical characteristics, meteorological and hydrographic data, the marine ecosystem, and the potential for fouling and pollution is necessary to design an appropriate water intake. 

The number of organisms affected by entrainment and impingement varies depending on the volume and velocity of the feed water and the use of mitigation measures developed to minimize their impact. Seasonal variations in migration or growth are also factors [103]. It is established that the water intake does not pose a threat to large marine species; however, smaller species such as phytoplankton and zooplankton may be harmed [104]. To minimize possible impacts, the water intake is located higher than the seabed and has large openings covered with mesh to reduce flow velocity and avoid impinging on small organisms such as larvae and eggs.

Brine disposal is the biggest concern for desalination, as it poses risks to marine ecosystems. Increased salinity, temperature, and the release of harmful substances, including heavy metals, scale inhibition additives, bio-fouling agents, antifoaming agents, coagulants/flocculants, cleaning chemicals, chlorine, and bisulfite [105,106,107,108,109] are the major issues. The degree of environmental impact caused by brine discharge depends on the flow rate and properties of the brine, the dilution rate, the physical, chemical, and biological properties of the receiving water body, and the discharge method. It is recognized that in a well-mixed environment, negative impacts are limited to a few hundred meters from the discharge point. In shallow and/or semi-enclosed bays, the impacts are more prominent [110]. 

Desalination plants located within 1–10 km from the shoreline typically discharge brine directly into the sea, as it is the most cost-effective disposal technique. In contrast, inland desalination plants employ several other methods, including discharge into a sewer system, deep-well injection, evaporation ponds, and land applications. However, these techniques are not sustainable and are constrained by high capital costs [105]. 

As explained in Section 3.2, SWRO brine is typically discharged into coastal waters through designated outfalls located either on the shoreline or offshore. In the case of open discharge outfalls, the brine is mixed with seawater primarily by the discharge velocity and the high-intensity underwater currents in the area [111]. Alternatively, the co-discharge of desalination brine with wastewater effluent offers advantages such as accelerating the dissipation of both the wastewater and the brine, as well as reducing the concentrations of metals, organics, and pathogens originating from the effluent in the combined stream. On the other hand, sub-sea diffuser systems offer greater dilution and mixing of the brine with seawater than open channel outfalls. The mixing in this discharge method, which involves multiple ports or a rosette pattern, is determined by various factors such as the discharge velocity, diffuser system design (including the discharge angle, subsurface depth, number and spacing between nozzles), brine salinity, and sea currents [75,112]. 

To evaluate the environmental impact of brine discharge on the receiving marine ecosystem, ongoing monitoring programs are implemented, which involve comparing the chemical and biological aspects pre- and post-operation of the desalination plant. The brine produced by SWRO is typically twice as salty as seawater, resulting in greater density and causing it to sink to the seafloor near the outlet, a phenomenon commonly referred to as the “plume effect”. The plume effect can be mitigated by discharging the brine into a strong sea current to promote effective mixing. Jet discharge, which involves directing the brine at an upward angle, can also reduce the spatial range and intensity of the plumes and improve mixing. Finally, dilution of the brine before discharge, such as by using cooling water from power stations, can be an effective approach to minimize the impact of brine discharge on the marine environment [113,114].

**Figure 3:**
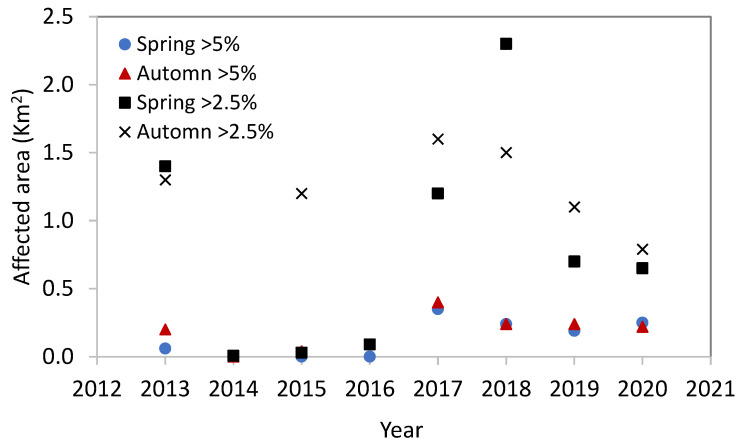
The area affected by the saline brine for ∆S > 2.5 and 5.0% (data from [115]).

The localized salinity effect of the brine discharge, as a result of the use of open outfalls and sub-sea multiport diffuser systems, is evident from global monitoring programs. A few examples are listed as follows. For instance, since the commencement of the SWRO Ashkelon desalination plant in Israel in 2006, instances of excessive salinity levels (i.e., ∆S) above the natural background have been recorded only twice: in the fall of 2017 and again in the fall of 2020, with ∆S levels reaching 12.5%. Over the period spanning 2013 to 2020, the area of the seabed in which ∆S levels exceeded 5% was limited to 0–0.4 km^2^ surrounding the brine outlet. Similarly, ∆S > 2.5% were measured in an area of 0–2.3 km^2^ (as shown in Figure 3). The Ashkelon desalination plant utilizes an open outfall system with three discharge points, all of which are located within 400 m of each other. Notably, the plant’s brine is mixed with the cooling water from the adjacent power stations as well as the brine from three inland BWRO desalination plants [115].

The outfalls of the adjacent Sorek and Palmachim SWRO desalination plants in Israel are located approximately 850 m apart, at a depth of 20 m. Given the proximity of the two outfalls, monitoring efforts have been implemented to assess the overall impact of brine discharge in the area. The Sorek outfall employs four diffusers, spaced 2.5 m apart in an alternate pattern and located 4 m above the seabed. The brine is released at a velocity of 4.0 m/s at a 45° upward angle. The Palmachim plant utilizes three risers situated 6 m above the seabed and spaced 6 m apart to discharge brine at a velocity of 1.4 m/s at a 45° upward angle. Results from monitoring efforts indicated a very localized ∆S > 5% close to the outfalls and near the seabed. The area of salinity that exceeded the background level by 1% (0.4 g/L) ranged from 3–24 km^2^. It should be noted that a 0.4 g/L variation in salinity is within the natural annual variability range observed in coastal waters [116]. 

In Spain, localized impacts of the salinity plume have also been monitored, as reported in [117,118,119]. This region hosts 50 SWRO and roughly 100 small BWRO desalination plants scattered throughout the Mediterranean Arc and the Canary Islands [120,121]. It is noteworthy that the management of brine discharge in Spain has been adapted based on the monitoring of marine ecosystems, to ensure effective mixing of the brine and to minimize any potential environmental impacts.

Most desalination plants in the countries bordering the Persian (Arabian) Gulf discharge brine via surface nearshore outfalls, leading to an average annual basin salinity increase of approximately 1% due to salt buildup [122]. This trend is particularly pronounced in the Persian Gulf region, which is characterized by low precipitation, limited freshwater input from land, and a high rate of evaporation, owing to its shallow, semi-enclosed sea with an average depth of 35 m [113]. The southwestern Gulf region surrounding Bahrain and the southern coast of Saudi Arabia is particularly sensitive to salinity, exhibiting a salt buildup of around 11%. It is important to note, however, that the Gulf’s freshwater sink from seawater desalination is considerably smaller than its freshwater sink from evaporation [122].

The Carlsbad desalination plant in the USA, with a capacity of 70 Mm^3^/year, discharges brine through an open channel outfall located approximately 50 m offshore. The discharge consists of a 1-to-10 ratio of brine to cooling water from the adjacent power station. Monitoring studies conducted before and after the plant’s operation indicate that the salinity of the surface water 50–1000 m from the outfall did not differ significantly. However, the salinity of near-seabed water in the same region increased by 1.1 g/L post-operation compared to pre-operation levels. Nevertheless, the mean values did not exceed the regulatory limit of two units over the background [123].

The power station’s cooling water increases the marine environment’s temperature, whereas the SWRO brine temperature remains ambient. The temperature plume distribution primarily depends on the power station’s operating conditions, such as the number of operating units, the level of electricity production, and the rate of cooling water discharge. Additionally, environmental conditions, especially wind velocity, also affect the temperature plume distribution. At the Ashkelon SWRO desalination plant during 2013–2020, the maximum values of ΔT (the difference between measured and natural background temperatures) were within 500 m of the outfall. ΔT was lower than 5–6 °C at about 1 km from the outfall [115]. The impacted area due to temperature increase (by 0.3–0.7 °C) was smaller than the area affected by salinity due to brine discharge from the Sorek and Palmachim desalination plants [116]. A temperature increase of 1–2 °C was measured in both surface and bottom waters around the outfall of the Carlsbad SWRO desalination plant [123], whereas no change in temperature was measured in the Sydney SWRO desalination plant located in Kurnell, New South Wales, Australia [124].

The water quality parameters in the area surveyed around Ashkelon’s desalination plant were found to be within the range of their natural concentrations, indicating that they were not affected by the brine discharge. These parameters include turbidity, dissolved oxygen, total suspended solids, nutrients, total organic carbon (TOC), particulate heavy metals, and particulate iron. However, elevated concentrations of total organic phosphorus (TOP), originating from the use of phosphonate-based antiscalants, were detected. Nevertheless, the additional TOP did not significantly increase the amount of phosphate in the surveyed area. Within a 500 m radius of the brine outfall, an increase in nitrate and silicic acid loading was observed between 2011 and 2020. This is attributed to a gradual rise in the concentrations of silicic acid and nitrate in the brine of inland BWRO desalination plants, which are mixed with the brine from the Ashkelon SWRO desalination plant [115].

The levels of pH, turbidity, suspended particulate matter, nitrate, nitrite, ammonium, total nitrogen, phosphate, TOC, and silicic acid in the vicinity of the Sorek and Palachim desalination plants were found to be within the natural range and in compliance with regulatory guidelines [116]. Similar results were obtained for the SWRO Carlsbad desalination plant [123].

The tolerance of marine species to salinity varies greatly depending on the salinity level and the duration of exposure. Salinities elevated by 10% above ambient levels do not affect relative abundances or growth rates, but they can alter community structures. At the SWRO Ashkelon plant, no significant impact on algae biomass was observed during the surveyed years. The infaunal community (i.e., number of specimens and species) was affected only in a narrow band of shallow water (up to 5 m deep) extending up to 600 m from the outlet and 500 m from shore [115]. In Sorek and Palmachim, there was no impact from brine discharge on the infauna beyond 200 m from the outfalls [116].

The benthic organisms inhabiting the seafloor around the desalination plants located along the Mediterranean coastline of Spain remain unaffected, despite the seasonal variation in the distribution of the salinity plume [119]. The minimal direct impact of the brine discharge from the Carlsbad SWRO desalination plant on the ecology of benthic organisms was observed [123]. In the Sydney SWRO desalination plant, a localized redistribution of species occurred over a small area within 100 m of the outfall, likely the result of changes to water flow hydrodynamics [124,125]. Nonetheless, no large or persistent loss of biodiversity was reported [126]. Fish species have been observed to be attracted to areas of brine discharge, as noted in several studies [117,124,125]. In addition, the reduction in fishing activity near the Valdelentisco SWRO desalination plant (Murcia, Spain), resulting from the installation of diffusers forming a marine reserve, has been associated with an increase in fish abundance [127]. It should be noted, however, that fish tend to avoid waters with salinities above background levels [128]. To summarize, long-term marine environment monitoring programs worldwide indicate that a well-designed brine discharge can limit the effects of salinity on the local area next to the discharge and even increase local fish abundance and species richness.

End-of-life RO membranes are solid waste that accumulate in landfills worldwide. A proposed sustainable solution is to recycle these aged RO membranes through oxidative treatments, which allows for their reuse in applications such as NF, UF, MF [129,130], membrane distillation [131], ED, and membrane biofilm reactor processes [132]. Nonetheless, the recycled filtration membranes may develop a high-pressure drop and therefore require frequent cleaning [133].

## 4. Water Reclamation

### 4.1. Overview

The process of transforming municipal and/or industrial wastewater into water suitable for reuse is known as water reclamation. This process offers a range of benefits, including improved water security, sustainability, and resilience, as well as alternative sources of water. Reclaimed water finds applications in a variety of settings, including agricultural and landscape irrigation, industrial processes (i.e., power plants, refineries, mills, and factories), potable water supplies, groundwater supply management, the creation of artificial lakes, and the restoration of inland or coastal aquifers and ecosystems.

It is important to note that while the discharge of treated wastewater (known as effluent) into streams may impair ecological communities and water quality, it can also help preserve aquatic habitats and restore flow in regions where water resources are limited. It has been estimated that the agricultural use of reclaimed water leads to a saving of 1.7 tons of CO_2_-eq.ha of carbon [134]. 

**Figure 4:**
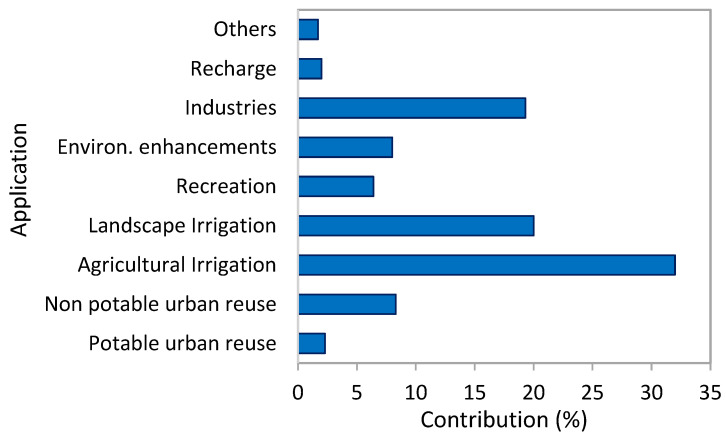
Major applications for water reuse and their contributions (data form [138]).

Jones et al. [135] estimated that 63% of global wastewater production is collected and 52% of it is treated, suggesting that 48% of the world’s wastewater is discharged untreated into the environment. This value represents a significant decrease from the previous estimate of around 80%. Untreated municipal wastewater has been identified as the most hazardous to water ecosystems due to the large amounts of nutrients and organic content [136]. Currently, only about 20% of the world’s wastewater is being reused for various applications [134,135], as illustrated in Figure 4. 

The production, collection, and treatment of wastewater are influenced by various factors, including geographical location and economic development. The reuse of treated wastewater is most prevalent in the Middle East and North Africa (15%) and Western Europe (16%) [135]. In Cyprus and Malta, the reuse of wastewater is 90% and 60%, respectively. Conversely, in Greece, Italy, and Spain, only 5–12% of treated wastewater is reused [136]. In Israel, 93% of wastewater is centrally treated, and 86% of it is repurposed for agricultural use. The use of reclaimed wastewater can account for up to 40–50% of Israel’s total agricultural water needs, particularly during periods of recurring drought when freshwater allocations are reduced to match the natural recharge of surface and groundwater resources [137].

Seawater desalination plants are typically located along the coast, while wastewater treatment plants (WWTPs) are situated on the outskirts of cities to facilitate the transport of the treated product to inland locations. The cost of desalinated wastewater leaving the plant is roughly equivalent to the cost of transporting clean water over a distance of 200 km [86], underscoring the importance of optimal plant location. By developing appropriate infrastructure, reclaimed water can be pumped inland to meet the demands of urban and agricultural sectors. The quality of the reclaimed water is higher when desalinated water supplies urban demands, as the salinity of the wastewater before treatment is significantly lower than that of natural hard water. Consequently, the reclaimed water has lower total dissolved solids (TDS), thereby reducing the problems associated with the salinity of reclaimed water [139].

Contrary to public perception [140,141,142], advanced technologies make it possible to produce potable water from effluents through desalination using RO or NF, or by mixing it with an environmental buffer such as rivers, lakes, or groundwater [143,144]. However, it is important to note that in hot climate regions, the water in storage lakes must be utilized frequently to prevent the accumulation of salt caused by significant evaporation rates. The use of flexible covered surfaces, which can also be equipped with solar collectors to generate solar energy, can help to minimize this issue.

Compared to alternative sources of irrigation water, appropriately treated reclaimed water does not pose an increased risk of produce-related illnesses or outbreaks [145]. Reclaimed water is primarily employed for agricultural irrigation, which accounts for more than 30% of the market [146]. The potential advantages, drawbacks, and obstacles associated with the utilization of reclaimed wastewater for agricultural purposes have been reviewed elsewhere [134,145,146,147]. High-quality effluent permits unrestricted reuse. Criteria governing permissible levels of salt, pollutants, metals, and other contaminants in reclaimed water differ by country. A snapshot of regulated parameters across countries is listed in Table 2.

Municipal wastewater, industrial process and cooling water, storm water, agricultural runoff, and return flows are among the water sources that can be treated for reuse. To meet “fit-for-purpose specifications” for specific end uses while complying with public health, environmental protection, and specific user needs [152], these waters undergo sufficient treatment. Often, the energy consumption is significantly lower when compared to a full treatment process required to comply with potable standards and centralized distribution for all uses when the specific reclamation goals are considered [153].

The economics of water reuse are highly dependent on the specific site. The cost of secondary effluent water from WWTPs ranges from 0.15 to 0.30 US $/m^3^, while reclaimed WWTP water costs between 0.25 and 0.50 US $/m^3^. It is essential to note that in the face of diminishing freshwater sources, escalating scarcity, and burgeoning demand, the cost factor becomes less significant [153].

Water reclamation utilizing membrane technologies is proving to be a crucial factor in enhancing the available water resources and achieving water sustainability. To achieve these goals, effective water use and reuse strategies are necessary [151]. In wastewater treatment, membrane technology is employed to eliminate salts and solids. Typically, UF MF is utilized for solids removal, while RO is used for salt removal. Membrane bioreactors (MBRs) and gravity-driven membrane (GDM) bioreactors are alternatives to conventional activated sludge (CAS) processes. These technologies exhibit superior pollutant retention capabilities and less fouling propensity.

The NEWater venture in Singapore is a successful example of membrane-based wastewater treatment. The process recycles treated used water into ultra-clean, high-grade reclaimed water, which is utilized in the microelectronics industry, while a portion is mixed with natural water for domestic consumption [154]. To date, five plants supply up to 40% of Singapore’s water needs. The NEWater production process involves filtration with MF or UF membrane, followed by desalination using RO, and final disinfection with ultraviolet light [154].

Several papers have provided a comprehensive review of the history, development, applications, and challenges of water reclamation using membrane technologies in the last five years [45,138,144,154,155,156,157,158,159,160,161]. The following sections provide an overview of the primary membrane-based wastewater treatment technologies, which include UF, MF, RO, MBR, and GDM.

### 4.2. Microfiltration and Ultrafiltration 

Microfiltration and ultrafiltration employ the sieving mechanism to remove particles. Microfiltration removes high molecular weight organic materials, suspended solids, colloids, and bacteria. Ultrafiltration removes particles, colloids, bacteria, protozoa, and viruses better than MF due to its smaller pores [138]. It is capable of removing all coliforms, reducing the bacterial count by 3-6 logs, the viral count by 2–7 logs, and eliminating more than 6 logs of protozoan cysts and oocysts [154]. Recent review articles on UF and MF can be found elsewhere [138,162,163].

Both MF and UF are limited in their ability to remove phosphorus, nitrates, ammonium, and micro-pollutants (MPs), but they are effective at removing residual organic matter [164]. However, the use of UF/MF processes for wastewater reclamation is restricted by membrane fouling caused by organic matter in the effluent. The fouling is primarily caused by pore blocking and cake filtration. Various treatments, such as sedimentation, adsorption, flocculation, and coagulation, have been used to reduce fouling by decreasing the organic content in the effluent or altering its composition and properties. Nevertheless, fouling is unavoidable, particularly in the hydrophilic fractions of the organic compounds [165].

### 4.3. Reverse Osmosis and Nanofiltration

Reverse osmosis membranes have been shown to significantly reduce total dissolved solids, heavy metals, MPs, viruses, bacteria, and other dissolved contaminants. Recommended practices for applying RO for wastewater reclamation to prevent rapid membrane fouling and thus reduce high system maintenance costs and significant downtime are the use of UF or MF pre-treatment to remove colloids and solids, maintaining a chloramine residual to prevent bio-growth, proper selection of antiscalant, limiting the RO recovery rates to prevent membrane scaling, and using membranes that minimize organic fouling [166].

RO and NF systems have shown effectiveness in removing endocrine-disrupting and pharmaceutically active compounds to levels below detection levels (<25 ng/L) [167]. The removal of MPs through RO or NF membranes is dependent on several factors, including the membrane properties, characteristics of the pollutants, electrolytes, solute characteristics, operating conditions, and membrane fouling. The mechanism of removing micro-pollutants by membrane filtration is primarily through size exclusion, especially for non-charged MPs, although other processes such as adsorption due to hydrophobic interactions and hydrogen bonding, electrostatic repulsion of charged MPs, and adsorption on the fouling layer can also play a role [168]. The presence of dissolved organic carbon and membrane fouling can modify the membrane surface characteristics and pore size, which may lead to increased adsorption of micro-pollutants [168,169]. 

### 4.4. Membrane Bioreactors—MBR

The MBR process combines biological treatment and UF membrane separation to retain suspended solids and maintains a high biomass concentration within the bioreactor. It can be operated aerobically or anaerobically with alternating phases to enhance microbial nitrification followed by denitrification [170]. MBRs are characterized by high volumetric loading, excellent effluent quality, reduced footprint, reduced sludge production, process flexibility concerning influent changes, and improved nitrification performance [171].

Compared to conventional activated sludge, MBRs offer several advantages. In CAS, wastewater is treated by microorganisms (i.e., activated sludge—AS) in an aeration tank, followed by the separation of treated water and activated sludge using a sedimentation tank or secondary clarifier. MBR provides better separation, leading to higher removal of MPs. MBRs have a higher solid retention time (SRT), greater biodiversity of microorganisms, and more opportunities for the adaptation of specific microorganisms to persistent compounds [167,168,172]. Table 3 lists the advantages and drawbacks of MBR in comparison to CAS. 

### 4.5. Gravity-Driven Membrane-GDM 

Gravity-driven membrane filtration involves UF membranes operated in a dead-end mode with biofilm allowed to develop on the membrane. This process does not require chemical or mechanical fouling and/or biofouling control, as ultra-low transmembrane pressures of <100 bar are used [172]. The biofilm layer contributes to the improvement of water quality by increasing the removal of various compounds, including humic acids, biopolymers, assimilable organic carbon (AOC), and algal toxins [173]. 

Integration with other processes, such as biofilm reactors, adsorption, and coagulation, may be used to improve GDM performance in terms of permeate flux and organic removal. At low capacities, the GDM process is often preferable to conventional UF. It provides robustness in remote locations and circumstances where process operators and electricity are not always available. A comprehensive review of the gravity-driven membrane filtration process was conducted by Pronk et al. (2019) [173].

## 5. Summary

Due to global climate change and population growth, water scarcity has become a critical issue, and it is necessary to take steps toward sustainable water resource management. One approach could be to implement measures, such as educating people on water conservation and appointing central-based water governance to oversee infrastructure and plan for extreme scenarios such as droughts and floods. Managing shared waters sustainably requires transboundary cooperation. Furthermore, addressing water scarcity requires exploring options such as improved water catchment, harvesting, conservation technologies, distribution infrastructure, irrigation, and agricultural practices, pollution control, investment in novel water technology, desalination, and water reclamation of domestic and industrial wastewater.

The scarcity of global water resources has made desalination one of the most important non-conventional water sources worldwide. However, one of the key challenges in implementing desalination is public concern over energy consumption and the environmental impacts of brine discharge. To address these challenges, various measures are taken, such as improving the efficiency and lifespan of membranes, optimizing the RO process, and utilizing renewable energy resources. The cost of desalinated water has significantly decreased over the years, with some high-capacity SWRO plants achieving costs as low as 0.28 US $/m^3^.

Long-term monitoring of desalination plants around the world has revealed that the impact of brine discharge is localized and minimal. It was established that a properly designed brine discharge can mitigate negative impacts on coastal and marine ecosystems, and may even contribute to an increase in local fish abundance and species richness. Hence, a significant increase in desalination production on a global scale is not expected to have adverse environmental impacts. 

Reclaimed water can be a viable and sustainable solution to water supply shortages, provided that it is integrated into a comprehensive water management strategy and meets health, safety, environmental, appearance, and economic criteria following local regulations. Membrane technologies, particularly UF and RO, have been instrumental in generating high-quality reclaimed water. These membranes are capable of removing a wide range of pollutants, including dissolved solids, viruses, bacteria, and low molecular weight organic contaminants. Membrane bioreactors have emerged as an efficient alternative to conventional activated sludge processes for treating wastewater. 

The threat to freshwater security is real and requires immediate action. With the implementation of practical engineering solutions and societal measures, it is possible to conserve natural water resources and turn alternative water resources into high-quality sources of water. These solutions can ensure a sustainable supply of freshwater for future generations and promote the overall well-being of society.

## Figures and Tables

**Table 1:** Typology of the potential benefits of transboundary water cooperation [53].

Economic	Societal	Environmental
Expand activity and productivity in agriculture, mining, energy, and nature	Reduced risk of water-related disasters	Preservation of resources
The reduced economic impact of water- related hazards such as floods and droughts	Employment and reduced poverty	Increase ecological integrity
Increased value of property	Improved access to services such as electricity and water supply	Reduced habitat degradation and biodiversity loss
Development of regional markets for goods, services, and labor	Strengthened scientific knowledge of water status	
Increase in cross-border investments	Strengthening of international law	
Development of transnational infrastructure networks	Increased geopolitical stability and strengthened diplomatic relations	
Joint initiatives and investments	Creation of a shared basin identity	
Avoided cost of conflicts		

**Table 2:** Discharge standards for municipal wastewater treatment plants in the US, EU, China, Israel, and Japan.

Parameter	US EPA [148]	EU [149,150]	China [149]	Israel [151]	Japan [149]
pH	6–9		6–9	6.5–8.5	5–9
COD (mg/L)	250	125	50	100	120
BOD_5_ (mg/L)	50	≤10	10	10	120
TSS (mg/L)	50	≤10	10	10	150
TN (mg/L)	50	10	15	25	60
NH_4_-N (mg/L)	1.0		5	10	
TP (mg/L)	2.0	1.0	0.5	5.0	8.0
E-coli (MPN/100 mL)	10	≤10		10	

Carbon Oxygen Demand (COD), Biochemical Oxygen Demand (BOD_5_), Total Suspended Solids (TSS), Total Nitrogen (TN), Ammonium as Nitrogen (NH_4_-N), and Total Phosphorus (TP).

**Table 3:** Advantages and disadvantages of MBR compared to CAS [172].

Advantages	Disadvantages
Smaller footprint	Membrane fouling
There are no limitations on the concentration of the mixed liquor suspended solids (MLSS) in the MBR, so the generation of waste AS is reduced. The maximum concentration of MLSS in CAS is around 5000 mg/L while the optimum level in MBR is around 8000–12,000 mg/L.	Higher capital and operational costs due to the cost of the membranes and antifouling strategies.
Fine control of the solid retention time (SRT) can be achieved in MBR due to the elimination of secondary sedimentation tanks.	Higher aeration requirement contributes to the increased foaming propensity.
Applicability of longer SRT in MBR (more than 20 days) in comparison to CAS (generally 5–15 days) provides higher effluent quality.	Higher power consumption during operation. In some cases, it is double the electricity consumption of CAS.
The generation of high-quality effluent due to a membrane separation eliminates the need for tertiary treatment.	

## Data Availability

Not applicable.

## References

[1] United Nations Sustainable Development Goals Clean Water and Sanitation. https://unstats.un.org/sdgs/report/2022/The-Sustainable-Development-Goals-Report-2022.pdf/.

[2] Ayboga E. Policy, and Impacts of Dams in the Euphrates and Tigris Basin. Mesopotamia Water Forum 6–8 April 2019, Sulaimani, Kurdistan Region of Iraq. https://www.savethetigris.org/wp-content/uploads/2019/01/Paper-Challenge-B-Dams-FINAL-to-be-published.pdf.

[3] Dhakal N., Salinas-Rodriguez S.G., Hamdani J., Abushaban A., Sawalha H., Schippers J.C., Kennedy M.D. (2022). Is Desalination a Solution to Freshwater Scarcity in Developing Countries?. Membranes.

[4] Domènech L. (2011). Rethinking water management: From centralised to decentralised water supplyand sanitation models. Doc. D’anàlisi Geogràfica.

[5] Kislev Y. The Water Economy of Israel. Taub Center for Social Policy Studies in Israel Jerusalem. Policy Paper No. 2011.15, Nov. 2011. https://openscholar.huji.ac.il/sites/default/files/agri_economics/files/38-2011_water_economy_taub_center.pdf.

[6] Bar-Nahum Z., Reznik A., Finkelshtain I., Kan I. (2021). Centralized water management under lobbying: Economic analysis of desalination in Israel. Ecol. Econ..

[7] Pugel K., Javernick-Will A., Peabody S., Nyaga C., Mussa M., Mekonta L., Dimtse D., Watsisi M., Buhungiro E., Mulatu T. (2021). Pathways for collaboratively strengthening water and sanitation systems. Sci. Total Environ..

[8] Galvez V., Rojas R., Bennison G., Prats C., Claro E. (2020). Collaborate or perish: Water resources management under contentious water use in a semiarid basin. Int. J. River Basin Manag..

[9] Sachidananda M., Webb D.P., Rahimifard S. (2016). A Concept of Water Usage Efficiency to Support Water Reduction in Manufacturing Industry. Sustainability.

[10] Bauer S., Wagner M. (2022). Possibilities and Challenges of Wastewater Reuse—Planning Aspects and Realized Examples. Water.

[11] Baba A., Tsatsanifos C., El Gohary F., Palerm J., Khan S., Mahmoudian S.A., Ahmed A.T., Tayfur G., Dialynas Y.G., Angelakis A.N. (2018). Developments in water dams and water harvesting systems throughout history in different civilizations. Int. J. Hydrol..

[12] Alim M.A., Rahman A., Tao Z., Samali B., Khan M.M., Shirin S. (2020). Suitability of roof harvested rainwater for potential potable water production: A scoping review. J. Clean. Prod..

[13] Rojas E.M., Ortiz E.A.D., Tafur C.A.M., García L., Oliva M., Briceño N.B.R. (2021). A Rainwater Harvesting and Treatment System for Domestic Use and Human Consumption in Native Communities in Amazonas (NW Peru): Technical and Economic Validation. Scientifica.

[14] Yildirim G., Alim M.A., Rahman A. (2022). Review of Rainwater Harvesting Research by a Bibliometric Analysis. Water.

[15] Xu J., Dai J., Wu X., Wu S., Zhang Y., Wang F., Gao A., Tan Y. (2023). Urban rainwater utilization: A review of management modes and harvesting systems. Front. Environ. Sci..

[16] Silva A.C.R.d.S., Bimbato A.M., Balestieri J.A.P., Vilanova M.R.N. (2022). Exploring environmental, economic and social aspects of rainwater harvesting systems: A review. Sustain. Cities Soc..

[17] Singh S., Yadav R., Kathi S., Singh A.N., Kathi S., Devipriya S., Thamaraiselvi K. (2022). Chapter 14-Treatment of harvested rainwater and reuse: Practices, prospects, and challenges. Cost Effective Technologies for Solid Waste and Wastewater Treatment.

[18] Pala G.K., Pathivada A.P., Velugoti S.J.H., Yerramsetti C., Veeranki S. (2021). Rainwater harvesting—A review on conservation, creation & cost-effectiveness. Mater. Today Proc..

[19] Semaan M., Day S.D., Garvin M., Ramakrishnan N., Pearce A. (2020). Optimal sizing of rainwater harvesting systems for domestic water usages: A systematic literature review. Resour. Conserv. Recycl. X.

[20] Słyś D., Stec A. (2020). Centralized or Decentralized Rainwater Harvesting Systems: A Case Study. Resources.

[21] Velasco-Muñoz J.F., Aznar-Sánchez J.A., Batlles-Delafuente A., Fidelibus M.D. (2019). Rainwater Harvesting for Agricultural Irrigation: An Analysis of Global Research. Water.

[22] Yannopoulos S., Giannopoulou I., Kaiafa-Saropoulou M. (2019). Investigation of the Current Situation and Prospects for the Development of Rainwater Harvesting as a Tool to Confront Water Scarcity Worldwide. Water.

[23] Malik R., Giordano M., Sharma V. (2014). Examining farm-level perceptions, costs, and benefits of small water harvesting structures in Dewas, Madhya Pradesh. Agric. Water Manag..

[24] Basel B., Quiroz N.H., Herrera R.V., Alonso C.S., Hoogesteger J. (2020). Bee mietii rak rkabni nis (The people know how to seed water): A Zapotec experience in adapting to water scarcity and drought. Clim. Dev..

[25] Basel B., Hoogesteger J., Hellegers P. (2022). Promise and paradox: A critical sociohydrological perspective on small-scale managed aquifer recharge. Front. Water.

[26] Seddon N., Chausson A., Berry P., Girardin C.A.J., Smith A., Turner B. (2020). Understanding the value and limits of nature-based solutions to climate change and other global challenges. Philos. Trans. R. Soc. B Biol. Sci..

[27] Pamidimukkala A., Kermanshachi S., Adepu N., Safapour E. (2021). Resilience in Water Infrastructures: A Review of Challenges and Adoption Strategies. Sustainability.

[28] Butler D., Ward S., Sweetapple C., Astaraie-Imani M., Diao K., Farmani R., Fu G. (2016). Reliable, resilient and sustainable water management: The Safe & SuRe approach. Glob. Chall..

[29] Kang H. (2019). Challenges for water infrastructure asset management in South Korea. Water Policy.

[30] Liu Q., Yang L., Yang M. (2021). Digitalisation for Water Sustainability: Barriers to Implementing Circular Economy in Smart Water Management. Sustainability.

[31] Taormina R., Galelli S., Tippenhauer N.O., Salomons E., Ostfeld A. (2017). Characterizing Cyber-Physical Attacks on Water Distribution Systems. J. Water Resour. Plan. Manag..

[32] FAO (2020). The State of Food and Agriculture 2020, Overcoming Water Challenges in Agriculture.

[33] Bwambale E., Abagale F.K., Anornu G.K. (2021). Smart irrigation monitoring and control strategies for improving water use efficiency in precision agriculture: A review. Agric. Water Manag..

[34] Levidow L., Zaccaria D., Maia R., Vivas E., Todorovic M., Scardigno A. (2014). Improving water-efficient irrigation: Prospects and difficulties of innovative practices. Agric. Water Manag..

[35] Bertule M., Appelquist L.R., Spensley J., Trærup S.L.M., Naswa P. (2018). Climate Change Adaptation Technologies for Water: A Practitioner’s Guide to Adaptation Technologies for Increased Water Sector Resilience.

[36] Tal A. (2016). Rethinking the sustainability of Israel’s irrigation practices in the Drylands. Water Res..

[37] Sauvé S., Desrosiers M. (2014). A review of what is an emerging contaminant. Chem. Cent. J..

[38] Zhao L., Deng J., Sun P., Liu J., Ji Y., Nakada N., Qiao Z., Tanaka H., Yang Y. (2018). Nanomaterials for treating emerging contaminants in water by adsorption and photocatalysis: Systematic review and bibliometric analysis. Sci. Total Environ..

[39] Rathi B.S., Kumar P.S., Show P.-L. (2020). A review on effective removal of emerging contaminants from aquatic systems: Current trends and scope for further research. J. Hazard. Mater..

[40] Sivaranjanee R., Kumar P.S. (2021). A review on remedial measures for effective separation of emerging contaminants from wastewater. Environ. Technol. Innov..

[41] Shahid M.K., Kashif A., Fuwad A., Choi Y. (2021). Current advances in treatment technologies for removal of emerging contaminants from water—A critical review. Coord. Chem. Rev..

[42] Kumar R., Qureshi M., Vishwakarma D.K., Al-Ansari N., Kuriqi A., Elbeltagi A., Saraswat A. (2022). A review on emerging water contaminants and the application of sustainable removal technologies. Case Stud. Chem. Environ. Eng..

[43] Varsha M., Kumar P.S., Rathi B.S. (2022). A review on recent trends in the removal of emerging contaminants from aquatic environment using low-cost adsorbents. Chemosphere.

[44] Morin-Crini N., Lichtfouse E., Fourmentin M., Ribeiro A.R.L., Noutsopoulos C., Mapelli F., Fenyvesi É., Vieira M.G.A., Picos-Corrales L.A., Moreno-Piraján J.C. (2022). Removal of emerging contaminants from wastewater using advanced treatments. A review. Environ. Chem. Lett..

[45] Sengupta A., Jebur M., Kamaz M., Wickramasinghe S.R. (2022). Removal of Emerging Contaminants from Wastewater Streams Using Membrane Bioreactors: A Review. Membranes.

[46] Priyadarshini M., Das I., Ghangrekar M.M., Blaney L. (2022). Advanced oxidation processes: Performance, advantages, and scale-up of emerging technologies. J. Environ. Manag..

[47] Kumar V., Bilal M., Ferreira L.F.R. (2022). Editorial: Recent Trends in Integrated Wastewater Treatment for Sustainable Development. Front. Microbiol..

[48] Söderholm P. (2020). The green economy transition: The challenges of technological change for sustainability. Sustain. Earth.

[49] Ahmed F., Johnson D., Hashaikeh R., Hilal N. (2023). Barriers to Innovation in Water Treatment. Water.

[50] Süsser D. (2020). Accelerating Cleantech Commercialization in Israel: Green Innovation as a Catalyst for Sustainable Development.

[51] Hao J. (2018). A Study of Cleantech Innovations in the Israeli Entrepreneurial Ecosystem. MIT Reap. https://reap.mit.edu/assets/Junli-Final.pdf.

[52] Petersen-Perlman J.D., Veilleux J.C., Wolf A.T. (2017). International water conflict and cooperation: Challenges and opportunities. Water Int..

[53] UNECE (2015). Policy Guidance Note on the Benefits of Transboundary Water Cooperation: Identification, Assessment, and Communication. Convention on the Protection and Use of Transboundary Watercourses and International Lakes. United Nations Economic Commission for Europe (UNECE). https://unece.org/environment-policy/publications/policy-guidance-note-benefits-transboundary-water-cooperation.

[54] CADRI Partnership (2020). Good Practices on Transboundary Water Resources Management and Cooperation. CADRI Partnership under the Leadership of FAO with Inputs from UN Environment and the United Nations Economic Commission for Europe. https://www.cadri.net/system/files/2021-09.

[55] Mohammed I.N., Bolten J.D., Souter N.J., Shaad K., Vollmer D. (2022). Diagnosing challenges and setting priorities for sustainable water resource management under climate change. Sci. Rep..

[56] UN World Development Report 2022. Groundwater, Making the Invisible Visible. https://www.unesco.org/reports/wwdr/2022/en.

[57] Shehata N., Egirani D., Olabi A., Inayat A., Abdelkareem M.A., Chae K.-J., Sayed E.T. (2023). Membrane-based water and wastewater treatment technologies: Issues, current trends, challenges, and role in achieving sustainable development goals, and circular economy. Chemosphere.

[58] Curto D., Franzitta V., Guercio A. (2021). A Review of the Water Desalination Technologies. Appl. Sci..

[59] Najim A. (2022). A review of advances in freeze desalination and future prospects. Npj Clean Water.

[60] Bo Z., Huang Z., Xu C., Chen Y., Wu E., Yan J., Cen K., Yang H., Ostrikov K. (2022). Anion-kinetics-selective graphene anode and cation-energy-selective MXene cathode for high-performance capacitive deionization. Energy Storage Mater..

[61] Zhao X., Wei H., Zhao H., Wang Y., Tang N. (2020). Electrode materials for capacitive deionization: A review. J. Electroanal. Chem..

[62] WHO (2017). Guidelines for Drinking-Water Quality: First Addendum.

[63] Vera A., Bastida F., Patiño-García M., Moreno J.L. (2023). The effects of boron-enriched water irrigation on soil microbial community are dependent on crop species. Appl. Soil Ecol..

[64] Jacob C. (2007). Seawater desalination: Boron removal by ion exchange technology. Desalination.

[65] Landsman M.R., Lawler D.F., Katz L.E. (2020). Application of electrodialysis pretreatment to enhance boron removal and reduce fouling during desalination by nanofiltration/reverse osmosis. Desalination.

[66] Milne N.A., O’Reilly T., Sanciolo P., Ostarcevic E., Beighton M., Taylor K., Mullett M., Tarquin A.J., Gray S.R. (2014). Chemistry of silica scale mitigation for RO desalination with particular reference to remote operations. Water Res..

[67] Haidari A., Witkamp G., Heijman S. (2022). High silica concentration in RO concentrate. Water Resour. Ind..

[68] Matin A., Rahman F., Shafi H.Z., Zubair S.M. (2019). Scaling of reverse osmosis membranes used in water desalination: Phenomena, impact, and control; future directions. Desalination.

[69] Valavala R., Sohn J.-S., Han J.-H., Her N.-G., Yoon Y.-M. (2011). Pretreatment in Reverse Osmosis Seawater Desalination: A Short Review. Environ. Eng. Res..

[70] Charcosset C. (2022). Classical and Recent Developments of Membrane Processes for Desalination and Natural Water Treatment. Membranes.

[71] Hasson D., Semiat R. (2006). Scale Control in Saline and Wastewater Desalination. Isr. J. Chem..

[72] Cohen Y., Semiat R., Rahardianto A. (2017). A perspective on reverse osmosis water desalination: Quest for sustainability. AIChE J..

[73] Lesimple A., Ahmed F.E., Hilal N. (2020). Remineralization of desalinated water: Methods and environmental impact. Desalination.

[74] Shemer H., Hasson D., Semiat R. (2015). State-of-the-art review on post-treatment technologies. Desalination.

[75] Missimer T.M., Maliva R.G. (2018). Environmental issues in seawater reverse osmosis desalination: Intakes and outfalls. Desalination.

[76] Backer S.N., Bouaziz I., Kallayi N., Thomas R.T., Preethikumar G., Takriff M.S., Laoui T., Atieh M.A. (2022). Review: Brine Solution: Current Status, Future Management and Technology Development. Sustainability.

[77] Bello A.S., Zouari N., Da’Ana D.A., Hahladakis J.N., Al-Ghouti M.A. (2021). An overview of brine management: Emerging desalination technologies, life cycle assessment, and metal recovery methodologies. J. Environ. Manag..

[78] Al-Absi R.S., Abu-Dieyeh M., Al-Ghouti M.A. (2021). Brine management strategies, technologies, and recovery using adsorption processes. Environ. Technol. Innov..

[79] Soliman M.N., Guen F.Z., Ahmed S.A., Saleem H., Khalil M.J., Zaidi S.J. (2021). Energy consumption and environmental impact assessment of desalination plants and brine disposal strategies. Process. Saf. Environ. Prot..

[80] Voutchkov N., Semiat R., Li N.N., Ho W.S., Fane A.G., Matsuura T. (2008). Seawater Desalination, in Advanced Membrane Technology and Applications.

[81] Wang X.-N., Ma M.-Y., Pan X.-H., Hao J., Zhang C.-N. (2020). Quality of product water by three full-scale seawater reverse osmosis desalination in China. Desalin. Water Treat.

[82] Semiat R. (2010). Water Purification: Materials and Technologies. Encyclopedia of Materials: Science and Technology.

[83] Shokri A., Fard M.S. (2023). Techno-economic assessment of water desalination: Future outlooks and challenges. Process. Saf. Environ. Prot..

[84] Gao L., Yoshikawa S., Iseri Y., Fujimori S., Kanae S. (2017). An Economic Assessment of the Global Potential for Seawater Desalination to 2050. Water.

[85] Miller S., Shemer H., Semiat R. (2015). Energy and environmental issues in desalination. Desalination.

[86] Shemer H., Semiat R. (2017). Sustainable RO desalination—Energy demand and environmental impact. Desalination.

[87] Park J., Lee S. (2022). Desalination Technology in South Korea: A Comprehensive Review of Technology Trends and Future Outlook. Membranes.

[88] Chu S., Zhang S., Ma X., Li Y., Qiu D., Ge W., Kou L. (2022). Experimental Study on the Influence of Flexible Control on Key Parameters in Reverse Osmosis Desalination. IEEE Access.

[89] Himeur Y., Elnour M., Fadli F., Meskin N., Petri I., Rezgui Y., Bensaali F., Amira A. (2023). AI-big data analytics for building automation and management systems: A survey, actual challenges and future perspectives. Artif. Intell. Rev..

[90] Kurihara M. (2021). Current Status and Future Trend of Dominant Commercial Reverse Osmosis Membranes. Membranes.

[91] Kettani M., Bandelier P. (2020). Techno-economic assessment of solar energy coupling with large-scale desalination plant: The case of Morocco. Desalination.

[92] Wilf M. Fundamentals of RO-NF technology. Proceedings of the International Conference on Desalination Costing.

[93] Glueckstern P. History of desalination cost estimations. Proceedings of the International Conference on Desalination Costing.

[94] Wittholz M.K., O’Neill B.K., Colby C.B., Lewis D. (2008). Estimating the cost of desalination plants using a cost database. Desalination.

[95] Reddy K., Ghaffour N. (2007). Overview of the cost of desalinated water and costing methodologies. Desalination.

[96] Reddy K.V. (2008). Review and evaluation of desalination cost and costing methodologies. Int. J. Nucl. Desalin..

[97] Jones E., Qadir M., van Vliet M.T., Smakhtin V., Kang S.-M. (2019). The state of desalination and brine production: A global outlook. Sci. Total Environ..

[98] Lokiec F. Sustainable desalination: Environmental approaches. Proceedings of the International Desalination Association World Congress on Desalination and Water Reuse.

[99] Thi H.T.D., Pasztor T., Fozer D., Manenti F., Toth A.J. (2021). Comparison of Desalination Technologies Using Renewable Energy Sources with Life Cycle, PESTLE, and Multi-Criteria Decision Analyses. Water.

[100] El-Ghonemy A. (2012). RETRACTED: Water desalination systems Powered by Renewable energy sources, Review. Renew. Sustain. Energy Rev..

[101] Mohammad N., Ishak W.W.M., Mustapa S.I., Ayodele B.V. (2021). Natural Gas as a Key Alternative Energy Source in Sustainable Renewable Energy Transition: A Mini Review. Front. Energy Res..

[102] Safari A., Das N., Langhelle O., Roy J., Assadi M. (2019). Natural gas: A transition fuel for sustainable energy system transformation?. Energy Sci. Eng..

[103] Pankratz T., Missimer T.M., Jones B., Maliva R.G. (2015). Overview of Intake Systems for Seawater Reverse Osmosis Facilities. Intakes and Outfalls for Seawater Reverse-Osmosis Desalination Facilities: Innovations and Environmental Impacts.

[104] Musfique A., Rifat A. (2012). An Assessment of the environmental impact of brine disposal in the marine environment. Int. J. Mod. Eng. Res..

[105] Panagopoulos A., Haralambous K.-J., Loizidou M. (2019). Desalination brine disposal methods and treatment technologies—A review. Sci. Total Environ..

[106] Einav R., Harussi K., Perry D. (2003). The footprint of the desalination processes on the environment. Desalination.

[107] Ketsetzi A., Stathoulopoulou A., Demadis K.D. (2008). Being “green” in chemical water treatment technologies: Issues, challenges and developments. Desalination.

[108] Roberts D.A., Johnston E.L., Knott N.A. (2010). Impacts of desalination plant discharges on the marine environment: A critical review of published studies. Water Res..

[109] Palomar P., Losada I.J., Schorr M. (2011). Impacts of Brine Discharge on the Marine Environment. Modeling as a Predictive Tool. Chapter 13. Desalination Trends and Technologies.

[110] Alameddine I., El-Fadel M. (2007). Brine discharge from desalination plants: A modeling approach to an optimized outfall design. Desalination.

[111] Voutchkov N. (2011). Overview of seawater concentrate disposal alternatives. Desalination.

[112] Abessi O., Roberts P.J.W. (2014). Multiport diffusers for dense discharges. J. Hydraul. Eng..

[113] Omerspahic M., Al-Jabri H., Siddiqui S.A., Saadaoui I. (2022). Characteristics of Desalination Brine and Its Impacts on Marine Chemistry and Health, With Emphasis on the Persian/Arabian Gulf: A Review. Front. Mar. Sci..

[114] Frank H., Fussmann K.E., Rahav E., Bar Zeev E. (2019). Chronic effects of brine discharge from large-scale seawater reverse osmosis desalination facilities on benthic bacteria. Water Res..

[115] Monitoring of the Marine and Coastal Environment of the Rutenberg Power Station (2011). The Ashkelon Desalination Plant, the Brackish Water Desalination Plants, and the Power Station of Dorad Energy, Intel Kiryat Gat.

[116] Kress N., Shoham-Frider E., Lubinevsky H. (2020). Monitoring the Effect of Brine Discharge on the Marine Environment of the Palmachim and Sorek Desalination Plants.

[117] Sanchez J.L., Zarzo D. Environmental challenges on design, construction, and operation of desalination plants worldwide. TexasDesal 2017. Proceedings of the Developing a Drought-Proof Water Supply Conference.

[118] Arconada B., Delgado P., García Á. (2013). Minimizing environmental risks on constructing marine pipelines: Aguilas desalination plant. Desalin. Water Treat..

[119] Voutchkov N., Kaiser G., Stover R., Lienhart J., Awerbuch L. Sustainable management of desalination plant concentrate-desalination industry position. Proceedings of the International Desalination Association World Congress on Desalination and Water Reuse, IDAWC19.

[120] Morote Á.-F., Rico A.-M., Moltó E. (2017). Critical review of desalination in Spain: A resource for the future?. Geogr. Res..

[121] Pulido-Bosch A., Vallejos A., Sola F. (2019). Methods to supply seawater to desalination plants along the Spanish mediterranean coast and their associated issues. Environ. Earth Sci..

[122] Ibrahim H.D., Eltahir E.A.B. (2019). Impact of Brine Discharge from Seawater Desalination Plants on Persian/Arabian Gulf Salinity. J. Environ. Eng..

[123] Petersen K.L., Heck N., Reguero B.G., Potts D., Hovagimian A., Paytan A. (2019). Biological and Physical Effects of Brine Discharge from the Carlsbad Desalination Plant and Implications for Future Desalination Plant Constructions. Water.

[124] Clark G.F., Knott N.A., Miller B.M., Kelaher B.P., Coleman M.A., Ushiama S., Johnston E.L. (2018). First large-scale ecological impact study of desalination outfall reveals trade-offs in effects of hypersalinity and hydrodynamics. Water Res..

[125] Kelaher B.P., Clark G.F., Johnston E.L., Coleman M.A. (2020). Effect of Desalination Discharge on the Abundance and Diversity of Reef Fishes. Environ. Sci. Technol..

[126] Marine & Estuarine Monitoring Program-Detailed Design (Version 3) Preferred Project Report for Sydney’s Desalination Project (Sydney Water). https://www.sydneydesal.com.au/media/1092/marine-and-estuarine-monitoring-program.pdf.

[127] Gil-Meseguer E., Espin J.M.G., Bernabe-Crespo M.B. (2019). Desalination and water security in Southeastern Spain. J. Politi-Ecol..

[128] Iso S., Suizu S., Maejima A. (1994). The lethal effect of hypertonic solutions and avoidance of marine organisms in relation to discharged brine from a destination plant. Desalination.

[129] Seibel F.B., Giubel G.O.M., Brião V.B., Shabani M., Pontié M. (2021). End-of-life reverse osmosis membranes: Recycle procedure and its applications for the treatment of brackish and surface water. J. Appl. Res. Water Wastewater.

[130] Pontié M., Awad S., Tazerout M., Chaouachi O., Chaouachi B. (2017). Recycling and energy recovery solutions of end-of-life reverse osmosis (RO) membrane materials: A sustainable approach. Desalination.

[131] De Paula E.C., Gomes J.C.L., Amaral M. (2017). Recycling of end-of-life reverse osmosis membranes by oxidative treatment: A technical evaluation. Water Sci. Technol..

[132] Contreras-Martínez J., Sanmartino J., Khayet M., García-Payo M., Nayak S.K., Dutta K., Gohil J.M. (2022). Chapter 11-Reuse and recycling of end-of-life reverse osmosis membranes. Advancement in Polymer-Based Membranes for Water Remediation.

[133] Veza J.M., Rodriguez-Gonzalez J.J. (2003). Second use for old reverse osmosis membranes: Wastewater treatment. Desalination.

[134] Lahlou F.-Z., Mackey H.R., Al-Ansari T. (2022). Role of wastewater in achieving carbon and water neutral agricultural production. J. Clean. Prod..

[135] Jones E.R., van Vliet M.T.H., Qadir M., Bierkens M.F.P. (2021). Country-level and gridded estimates of wastewater production, collection, treatment and reuse. Earth Syst. Sci. Data.

[136] (2018). EU Policy on the Environment, Water Reuse. http://ec.europa.eu/environment/water/reuse.htm.

[137] Zaidi M.K. (2007). Wastewater Reuse–Risk Assessment, Decision-Making and Environmental Security.

[138] Yang J., Monnot M., Ercolei L., Moulin P. (2020). Membrane-Based Processes Used in Municipal Wastewater Treatment for Water Reuse: State-of-the-Art and Performance Analysis. Membranes.

[139] Shtull-Trauring E., Cohen A., Ben-Hur M., Tanny J., Bernstein N. (2020). Reducing salinity of treated waste water with large scale desalination. Water Res..

[140] Portman M.E., Vdov O., Schuetze M., Gilboa Y., Friedler E. (2022). Public perceptions and perspectives on alternative sources of water for reuse generated at the household level. J. Water Reuse Desalin..

[141] Furlong C., Jegatheesan J., Currell M., Iyer-Raniga U., Khan T., Ball A.S. (2019). Is the global public willing to drink recycled water? A review for researchers and practitioners. Util. Policy.

[142] Ormerod K.J. (2016). Illuminating Elimination: Public Perception and the Production of Potable Water Reuse. Wiley Interdiscip. Rev. Water.

[143] Tortajada C., Nambiar S. (2019). Communications on Technological Innovations: Potable Water Reuse. Water.

[144] Tang C.Y., Yang Z., Guo H., Wen J.J., Nghiem L.D., Cornelissen E. (2018). Potable Water Reuse through Advanced Membrane Technology. Environ. Sci. Technol..

[145] Partyka M.L., Bond R.F. (2022). Wastewater reuse for irrigation of produce: A review of research, regulations, and risks. Sci. Total Environ..

[146] Helmecke M., Fries E., Schulte C. (2020). Regulating water reuse for agricultural irrigation: Risks related to organic micro-contaminants. Environ. Sci. Eur..

[147] Chojnacka K., Witek-Krowiak A., Moustakas K., Skrzypczak D., Mikula K., Loizidou M. (2020). A transition from conventional irrigation to fertigation with reclaimed wastewater: Prospects and challenges. Renew. Sustain. Energy Rev..

[148] Owusu-Ansah E.D.-G.J., Sampson A., Amponsah S.K., Abaidoo R.C., Hald T. (2015). Performance, Compliance and Reliability of Waste Stabilization Pond: Effluent Discharge Quality and Environmental Protection Agency Standards in Ghana. Res. J. Appl. Sci. Eng. Technol..

[149] Lu X., Zhou B., Vogt R.D., Seip H.M., Xin Z., Ekengren Ö. (2016). Rethinking China’s water policy: The worst water quality despite the most stringent standards. Water Int..

[150] Regulation (EU) 2020/741 of the European Parliament and of the Council of 25 May 2020. https://climate-adapt.eea.europa.eu/en/metadata/guidances/regulation-on-minimum-requirements-for-water-reuse.

[151] Israeli Wastewater Quality Standards for Wastewater Treatment 2010. https://www.nevo.co.il/law_html/law01/500_306.htm.

[152] USEPA Basic Information about Water Reuse. https://www.epa.gov/waterreuse/basic-information-about-water-reuse.

[153] Capodaglio A.G. (2020). Fit-for-purpose urban wastewater reuse: Analysis of issues and available technologies for sustainable multiple barrier approaches. Crit. Rev. Environ. Sci. Technol..

[154] Bai Y., Shan F., Zhu Y.-Y., Xu J.-Y., Wu Y.-S., Luo X.-G., Wu Y.-H., Hu H.-Y., Zhang B.-L. (2020). Long-term performance and economic evaluation of full-scale MF and RO process—A case study of the changi NEWater Project Phase 2 in Singapore. Water Cycle.

[155] Wu J., Zhang Y., Wang J., Zheng X., Chen Y. (2021). Municipal wastewater reclamation and reuse using membrane-based technologies: A review. Desalin. Water Treat..

[156] Gurreri L., Tamburini A., Cipollina A., Micale G. (2020). Electrodialysis Applications in Wastewater Treatment for Environmental Protection and Resources Recovery: A Systematic Review on Progress and Perspectives. Membranes.

[157] Tibi F., Charfi A., Cho J., Kim J. (2020). Fabrication of polymeric membranes for membrane distillation process and application for wastewater treatment: Critical review. Process. Saf. Environ. Prot..

[158] Wu B., Kim J. (2020). Anaerobic Membrane Bioreactors for Nonpotable Water Reuse and Energy Recovery. J. Environ. Eng..

[159] Kharraz J.A., Khanzada N.K., Farid M.U., Kim J., Jeong S., An A.K. (2022). Membrane distillation bioreactor (MDBR) for wastewater treatment, water reuse, and resource recovery: A review. J. Water Process. Eng..

[160] Aslam A., Khan S.J., Shahzad H.M.A. (2021). Anaerobic membrane bioreactors (AnMBRs) for municipal wastewater treatment- potential benefits, constraints, and future perspectives: An updated review. Sci. Total Environ..

[161] Ang W.L., Mohammad A.W., Hilal N., Leo C.P. (2015). A review on the applicability of integrated/hybrid membrane processes in water treatment and desalination plants. Desalination.

[162] Kammakakam I., Lai Z. (2023). Next-generation ultrafiltration membranes: A review of material design, properties, recent progress, and challenges. Chemosphere.

[163] Anis S.F., Hashaikeh R., Hilal N. (2019). Microfiltration membrane processes: A review of research trends over the past decade. J. Water Process. Eng..

[164] Qu F., Wang H., He J., Fan G., Pan Z., Tian J., Rong H., Li G., Yu H. (2019). Tertiary treatment of secondary effluent using ultrafiltration for wastewater reuse: Correlating membrane fouling with rejection of effluent organic matter and hydrophobic pharmaceuticals. Environ. Sci. Water Res. Technol..

[165] Bartels C.R. (2006). Reverse osmosis membranes for wastewater reclamation. WaterWorld.

[166] Snyder S., Adham S., Redding A.M., Cannon F.S., Decarolis J., Oppenheimer J., Wert E.C., Yoon Y. (2007). Role of membranes and activated carbon in the removal of endocrine disruptors and pharmaceuticals. Desalination.

[167] Sreejon D., Nillohit M.R., Wan J., Khan A., Chakraborty T., Madhumita B.R., Farooq R., Ahmad Z. (2017). Micropollutants in Wastewater: Fate and Removal Processes. Physico-Chemical Wastewater Treatment and Resource Recovery.

[168] Comerton A.M., Andrews R.C., Bagley D.M., Hao C. (2008). The rejection of endocrine disrupting and pharmaceutically active compounds by NF and RO membranes as a function of compound and water matrix properties. J. Membr. Sci..

[169] Judd S. (2008). The status of membrane bioreactor technology. Trends Biotechnol..

[170] Banti D.C., Tsangas M., Samaras P., Zorpas A. (2020). LCA of a membrane bioreactor compared to activated sludge system for municipal wastewater treatment. Membranes.

[171] Al-Asheh S., Bagheri M., Aidan A. (2021). Membrane bioreactor for wastewater treatment: A review. Case Stud. Chem. Environ. Eng..

[172] Stoffel D., Rigo E., Derlon N., Staaks C., Heijnen M., Morgenroth E., Jacquin C. (2022). Low maintenance gravity-driven membrane filtration using hollow fibers: Effect of reducing space for biofilm growth and control strategies on permeate flux. Sci. Total Environ..

[173] Pronk W., Ding A., Morgenroth E., Derlon N., Desmond P., Burkhardt M., Wu B., Fane A.G. (2018). Gravity-driven membrane filtration for water and wastewater treatment: A review. Water Res..

